# Pain, Erythema, and Edema After Facial Lifting With Ultherapy Prime or Ultherapy Legacy—A Survey Study

**DOI:** 10.1111/jocd.70467

**Published:** 2025-09-18

**Authors:** Dissapong Panithaporn

**Affiliations:** ^1^ The Demis Clinic Bangkok Thailand

**Keywords:** edema, erythema, micro‐focused ultrasound, minimally invasive aesthetic intervention, pain, questionnaire

## Abstract

**Background:**

Micro‐focused ultrasound (MFU) can be used to achieve lifting of the brow, lifting of lax submental and neck areas, improving lines and wrinkles on the décolleté, through delivery of ultrasound on tiny points at different levels of the skin. This induces a wound repair reaction, which can achieve the tension and pulling effect of deep dermis and fascia.

**Aims:**

The objective of this study was to compare patient‐reported outcomes, including pain, erythema, and edema, following treatment with Ultherapy Prime and Ultherapy Legacy.

**Methods:**

A posttreatment questionnaire was used to collect data on pain, erythema, or edema after treatment with Ultherapy Prime and Ultherapy Legacy.

**Results:**

This study included 231 participants who underwent treatment with Ultherapy Legacy and Ultherapy Prime and 92 participants who underwent Ultherapy Prime as first‐time treatment. Participants reported significantly less pain, erythema, and edema after treatment with Ultherapy Prime, compared to treatment with Ultherapy Legacy, regardless of whether Ultherapy Prime was performed after Ultherapy Legacy or as first‐time treatment.

**Conclusion:**

Despite some limitations, e.g., a single‐center study performing unblinded treatment of participants, in the absence of a crossover design, participants consistently reported less pain, erythema, and edema after treatment with Ultherapy Prime, supporting its efficacy and safety in nonsurgical facial lifting. However, larger studies in more diverse geographical areas are needed to confirm these findings and rule out the impact of cultural differences on pain perception and expression.

## 1 Introduction

Wrinkles are a common indicator of aging, often prompting individuals to seek cosmetic treatment to restore a youthful appearance. Minimally invasive aesthetic interventions, including micro‐focused ultrasound (MFU), can mitigate the signs of aging [1]. Ultherapy (Merz North America Inc., Raleigh, NC, USA) utilizes micro‐focused ultrasound with visualization (MFU‐V) [2]. Ultherapy is US‐FDA‐cleared to achieve lifting of the brow, lifting of lax submental and neck areas, improving lines and wrinkles on the décolleté, and visualization of the dermal and subdermal tissue layers [3]. This technology delivers focused high‐energy ultrasound energy of 65°C–75°C on tiny points at different levels of the skin and induces a wound repair reaction, which can achieve the tension and pulling effect of deep dermis and fascia [2]. MFU is indicated in patients with mild to moderate skin laxity [4], and is contraindicated in patients with cystic acne, active infections, open wounds in the treatment area, and in pregnant patients [1, 4].

Previous research has assessed patient satisfaction following MFU. About half to two‐thirds of patients indicated they were satisfied or very satisfied with the results [5, 6]. Satisfaction was impacted by the region treated, the number of treatment locations, and the provider (consultant versus laser expert) [5]. Much less is known about the patients' perception of pain, erythema, and edema after MFU treatment. The purpose of this study was to compare patient‐reported outcomes, including pain, erythema, and edema, following treatment with the two MFU systems.

## 2 Methods

### 2.1 Study Design

This retrospective cross‐sectional survey was conducted as part of routine quality monitoring at The Demis Clinic, a privately owned dermatology clinic in Bangkok, Thailand. A convenience sample was drawn from consecutive patients who received MFU treatment using Ultherapy Prime between January and May 2025 (Merz North America Inc., Raleigh, NC, USA). The majority of patients had previously undergone Ultherapy Legacy treatment (Merz North America Inc., Raleigh, NC, USA) at the same clinic prior to 2025. Their previously recorded feedback was retrieved and directly compared with their responses following Ultherapy Prime treatment. For some patients, Ultherapy Prime was the first‐time MFU treatment.

Since patient feedback has routinely been collected during the introduction of new energy‐based devices, this study reflects real‐world clinical practice and did not interfere with the standard treatment workflow.

This survey study has been reported in line with the CROSS Guideline [7].

### 2.2 Questionnaire

The questionnaire consisted of three items questions, which were scored on a scale from 0 (no pain, erythema, or edema) to 10 (most severe). No personally identifiable data were collected. Each form was labeled with a unique study ID and completed in pen‐and‐paper format. The brief questionnaire was designed for completion within minutes during the patient's visit, requiring no additional appointment time.

Prior to full deployment, the form was pilot tested on the first five participants to ensure clarity. Minor wording adjustments were made based on feedback. All participants were briefly informed of the questionnaire's purpose, and participation was voluntary. Completion of the form was considered as implied informed consent.

As the data were anonymous, non‐interventional, and collected under routine clinical quality monitoring, the study was not submitted for institutional review board (IRB) approval.

A copy of the final questionnaire is provided in Appendix S1.

### 2.3 Participants

Participants enrolled were adults (18 years or older) of any gender who demonstrated a clinical need for a facelift.

All patients who underwent MFU treatment during the study period were invited to complete a brief posttreatment questionnaire as part of the clinic's routine quality monitoring process. The exact number of patients who declined participation was not recorded; therefore, the response rate could not be determined. Due to the short length and routine nature of the questionnaire, all participants completed the questionnaire in full. No missing data were observed.

### 2.4 Statistical Analysis

A paired *t* test was used to compare results after Ultherapy Legacy and Ultherapy Prime. Participant‐reported pain, erythema, and edema outcomes were divided into five groups: level 0 (none), levels 1–3 (mild), levels 4–6 (moderate), levels 7–9 (severe), level 10 (extreme).

## 3 Results

### 3.1 Respondent Characteristics

Enrollment of the Ultherapy Prime group took place from January to May 2025 and included 231 participants. These same participants previously underwent Ultherapy Legacy treatment, and their previously recorded feedback was retrieved. A further 92 participants were enrolled who had not been treated previously with Ultherapy Legacy.

Participant characteristics are shown in Table 1. Reasons for nonparticipation were not recorded. Given the limited size of the questionnaire, all participants fully completed it. As this was a random sample of patients visiting the Demis clinic, the sample can be considered representative of the target population.

**TABLE 1:** Participant characteristics.

	Both Legacy and Prime (*n* = 231)	Ultherapy Prime as 1st treatment (*n* = 92)
Gender		
Female, *n* (%)	195 (84.4%)	78 (84.8%)
Male, *n* (%)	36 (15.6%)	14 (15.2%)
Age in years, mean (SD)	44.0 (8.0)	
BMI, mean (SD)	20.8 (3.5)	21.3
Fitzpatrick skin type		
Type I, *n* (%)	24 (10.4%)	
Type II, *n* (%)	96 (41.6%)	
Type III, *n* (%)	88 (38.1%)	
Type IV, *n* (%)	23 (10.0%)	

Abbreviations: BMI, body mass index; SD, standard deviation.

### 3.2 Main Findings

Participants who underwent both treatments consistently reported less pain, less erythema, and less edema after treatment with Ultherapy Prime, compared to treatment with Ultherapy Legacy, as shown in Table 2.

**TABLE 2:** Pain, erythema, and edema after Ultherapy Legacy and Ultherapy Prime.

	Ultherapy Legacy		Ultherapy Prime		Difference		*p*
	Mean	SD	Mean	SD	Mean	SD	
Pain	5.59	2.36	2.75	1.93	2.84	1.84	< 0.001*
Erythema	3.23	2.17	1.61	1.61	1.63	1.62	< 0.001*
Edema	2.97	2.23	1.31	1.51	1.66	1.77	< 0.001*

*Note:* Paired *t* test.

Abbreviation: SD, standard deviation.

* Statistically significant difference.

After grouping of levels, treatment with Ultherapy Legacy was compared to consecutive treatment with Ultherapy Prime, as well as with Ultherapy Prime as first‐time treatment, as shown in Table 3. This confirms that reported pain, erythema, and edema levels are lower after Ultherapy Prime, regardless of whether after Ultherapy Legacy or as first‐time treatment.

**TABLE 3:** Comparison Ultherapy Prime after Ultherapy Legacy versus Ultherapy Prime as first‐time treatment.

Level	Legacy (*n* = 231)			Prime after Legacy (*n* = 231)			Prime (1st, *n* = 92)		
	Pain	Erythema	Edema	Pain	Erythema	Edema	Pain	Erythema	Edema
0	1 (< 1%)	21 (9%)	33 (14%)	23 (10%)	60 (26%)	84 (36%)	8 (8%)	21 (22%)	32 (33%)
1–3	51 (22%)	122 (53%)	120 (52%)	130 (56%)	142 (61%)	126 (55%)	48 (50%)	61 (64%)	56 (58%)
4–6	90 (39%)	63 (27%)	58 (25%)	70 (30%)	25 (11%)	18 (8%)	33 (34%)	10 (10%)	8 (8%)
7–9	81 (35%)	25 (11%)	19 (8%)	8 (3%)	4 (2%)	3 (1%)	7 (7%)	3 (3%)	0
10	8 (3%)	0	1 (< 1%)	0	0	0	0	1 (1%)	0

*Note:* Level 0 (none), levels 1–3 (mild), levels 4–6 (moderate), levels 7–9 (severe), level 10 (extreme).

## 4 Discussion

This study on 231 participants receiving MFU to mitigate the signs of aging showed significantly less pain, less erythema, and less edema after treatment with Ultherapy Prime, compared to treatment with Ultherapy Legacy. As seen in most studies on cosmetic treatment, males are underrepresented in this study. Because this is a consistent finding, it is not necessarily a limitation. The strength of this study is that treatment with Ultherapy Legacy and Ultherapy Prime was compared in the same participants, leading to a more accurate comparison than having different participants in both groups. However, this study also has some limitations. First of all, treatment was unblinded to participants, which may have led to a biased preference for the newer technology (“newer is better”). The data from Ultherapy Prime first‐time users show very similar outcomes in levels of pain, erythema, and edema, suggesting no bias was present in those who received Ultherapy Prime after Ultherapy Legacy. Secondly, although a large number of participants were included, the study was performed in a single center, limiting the generalizability of the findings. For instance, cultural differences can impact the perception and expression of pain [8], potentially leading to different outcomes in different geographical and cultural regions. Larger studies in more diverse geographical areas are needed to confirm these findings. These studies would also provide the ability to stratify results by BMI or Fitzpatrick skin type. Previous studies have shown that no change was detected in 54.5% of patients with a BMI above 30 but only in 12.2% of patients whose BMI was below 30 [9], while significantly higher Subject Global Aesthetic Improvement Scale (SGAIS) scores were observed among patients with a BMI of ≤ 25 kg/m^2^ [10]. Investigating the impact of BMI on perceived pain, erythema, and edema would be interesting. Similarly, an open‐label, nonrandomized trial in adults with Fitzpatrick skin types III to VI showed that, when performed by trained physicians, MFU is safe in patients with Fitzpatrick skin types III to VI [11]. While the results of this study suggest that Ultherapy Legacy and especially Ultherapy Prime are well tolerated, the study size did not allow stratification by skin type.

In conclusion, participants reported significantly lower pain, erythema, and edema scores after Ultherapy Prime, compared to the previous generation treatment (Ultherapy Legacy). These findings suggest that Ultherapy Prime provides a more comfortable posttreatment experience with fewer adverse effects, supporting its efficacy and safety in nonsurgical facial lifting. However, larger studies in more diverse geographical areas are needed to confirm these findings and rule out the impact of cultural differences on pain perception and expression.

## Author Contributions

Dissapong Panithaporn developed, wrote and approved this manuscript.

## Disclosure

Dr. Dissapong Panithaporn is self‐employed in the Demis Clinic, Bangkok, Thailand. No logistical and financial support for the execution of this study was received.

## Ethics Statement

All medical procedures described in this report were conducted in accordance with the Declaration of Helsinki.

## Consent

All participants were informed, and completion of the questionnaire was taken as consent to participate.

## Conflicts of Interest

The author declares no conflicts of interest.

## Supporting information


**Appendix S1:** jocd70467‐sup‐0001‐AppendixS1.pdf.

## Data Availability

The data presented in this report are available on request from the corresponding author.
