# Supplementary material for: Pain, Erythema, and Edema After Facial Lifting With Ultherapy Prime or Ultherapy Legacy—A Survey Study
Source: J Cosmet Dermatol. 2025 Sep 18;24(9):e70467. doi: 10.1111/jocd.70467 (PMC12445399; doi:10.1111/jocd.70467)
Supplement: Supplementary file 1 — Appendix S1: jocd70467‐sup‐0001‐AppendixS1.pdf. [file JOCD-24-e70467-s001.pdf]

Full Name_________________________________ Age________ years Gender_________

Weight________kg. Height________cm. Skin Tone TYPE 1 TYPE 2 TYPE 3 TYPE 4 TYPE 5 TYPE 6

Post-Ultherapy Evaluation – Please rate each item on a scale of 0 to 10

Treatment Date_____________

Question	Rating Scale										
	0	1	2	3	4	5	6	7	8	9	10
1. Pain during the procedure	No pain					Moderate pain					Most severe pain
2. Redness (erythema) on the skin immediately after the procedure	Normal skin					Moderate redness					Most severe redness
3. Swelling (edema) on the skin immediately after the procedure	No swelling					Moderate swelling					Most severe swelling

Previous Treatment

Question	Rating Scale										
	0	1	2	3	4	5	6	7	8	9	10
1. Pain during the procedure	No pain					Moderate pain					Most severe pain
2. Redness (erythema) on the skin immediately after the procedure	Normal skin					Moderate redness					Most severe redness
3. Swelling (edema) on the skin immediately after the procedure	No swelling					Moderate swelling					Most severe swelling

Full Name_________________________________ Age________ years Gender_________

Weight________kg. Height________cm. Skin Tone TYPE 1 TYPE 2 TYPE 3 TYPE 4 TYPE 5 TYPE 6

Post-Ultherapy Evaluation – Please rate each item on a scale of 0 to 10

Treatment Date_____________

Question	Rating Scale										
	0	1	2	3	4	5	6	7	8	9	10
1. Pain during the procedure	No pain					Moderate pain					Most severe pain
2. Redness (erythema) on the skin immediately after the procedure	Normal skin					Moderate redness					Most severe redness
3. Swelling (edema) on the skin immediately after the procedure	No swelling					Moderate swelling					Most severe swelling

Previous Treatment

Question	Rating Scale										
	0	1	2	3	4	5	6	7	8	9	10
1. Pain during the procedure	No pain					Moderate pain					Most severe pain
2. Redness (erythema) on the skin immediately after the procedure	Normal skin					Moderate redness					Most severe redness
3. Swelling (edema) on the skin immediately after the procedure	No swelling					Moderate swelling					Most severe swelling
